# Depressive states in healthy subjects lead to biased processing in frontal-parietal ERPs during emotional stimuli

**DOI:** 10.1038/s41598-023-44368-0

**Published:** 2023-10-11

**Authors:** Pengcheng Li, Mio Yokoyama, Daiki Okamoto, Hironori Nakatani, Tohru Yagi

**Affiliations:** 1 School of Environment and Society, Tokyo Institute of Technology, Tokyo, 152-8550 Japan; 2 School of Information and Telecommunication Engineering, Tokai University, Tokyo, 108-0074 Japan; 3 School of Engineering, Tokyo Institute of Technology, Tokyo, 152-8550 Japan

**Keywords:** Cognitive neuroscience, Depression

## Abstract

Subthreshold depressive (sD) states and major depression are considered to occur on a continuum, and there are only quantitative and not qualitative differences between depressive states in healthy individuals and patients with depression. sD is showing a progressively increasing prevalence and has a lifelong impact, and the social and clinical impacts of sD are no less than those of major depressive disorder (MDD). Because depression leads to biased cognition, patients with depression and healthy individuals show different visual processing properties. However, it remains unclear whether there are significant differences in visual information recognition among healthy individuals with various depressive states. In this study, we investigated the event-related potentials (ERPs) and event-related spectrum perturbation (ERSP) of healthy individuals with various depressive states during the perception of emotional visual stimulation. We show that different neural activities can be detected even among healthy individuals. We divided healthy participants into high, middle, and low depressive state groups and found that participants in a high depressive state had a lower P300 amplitude and significant differences in fast and slow neural responses in the frontal and parietal lobes. We anticipate our study to provide useful parameters for assessing the evaluation of depressive states in healthy individuals.

## Introduction

Major depressive disorder (MDD) is a mental illness accompanied by many depressive symptoms, including feelings of worthlessness, low mood, easy fatigue, and inability to concentrate, which can gravely affect people’s daily lives and their functioning in society^1^. Subthreshold depression (sD) and major depression are considered to occur on a continuum, and there are only quantitative and not qualitative differences between depressive states in healthy people and patients with MDD^2^. Qualitative differences refer to the different types of phenomena, such as psychological experience and depressive symptom. Quantitative differences, on the other hand, means that they differ in terms of how much depression is present, or how severe the symptoms are, but not in the type of symptoms that are present^3^. The depressive states lie on a single continuum or spectrum, where the main difference between mild, moderate, and severe depression is how severe or how many symptoms are, not the specific type or nature of the symptoms. Individuals with sD have a greater likelihood of developing MDD compared with individuals without sD^4^. sD is showing a progressively increasing prevalence and has a lifelong impact^5^, and the social and clinical impacts of sD are no less than those of MDD, with the higher prevalence of sD increasing its impact in terms of excess economic costs and mortality^6^. During the COVID-19 pandemic, the prevalence of both sub-threshold depression and major depressive disorder among university students was alarmingly high, underscoring the importance of early diagnosis and targeted interventions for this population^7^. Therefore, research into sD will contribute to a more detailed estimation of depressive states in healthy individuals, and thus to symptomatic treatment, and exploration of the neural mechanisms will help to explain the pathological mechanisms of depression.

The significant effect of depression on cognitive processes is reflected in the bias toward negative information. Previous research found that participants with MDD gaze longer at faces with negative expressions^8^ and focus more on negative self-evaluation words^9^. In terms of unconscious situations, participants with MDD lack an attentional bias toward negative emotions when unconscious compared with healthy participants, which would influence negative stimulus processing during consciousness^10^. Patients who are more depressed have a greater attentional bias toward emotional stimuli^11^, and this is reflected in the longer reaction time required to shift attention from emotional stimuli^12,13^. Such biased processing is also reflected in neural responses, including event-related potentials (ERPs) and event-related spectrum perturbation (ERSP), which present EEG oscillations at certain frequencies. ERP is an EEG feature with high temporal resolution obtained by averaging the neural activity of the cerebrum after a certain stimulus is presented^14^. Participants with MDD have ERPs that are significantly sensitive to negative pictures compared with healthy controls^15^, and they have a weaker frontal-parietal network, which is thought to be associated with impaired attentional selection, emotion regulation, and cognitive attentional control^8,16^. Sub-threshold subjects showed sharper ERP response and slower reaction time for emotional stimuli^17^. Depressed patients have significantly lower explicit self-esteem than healthy controls and that their central parietal P300 amplitude is attenuated in implicit self-esteem versus positive contexts^18^. ERSP shows oscillations in the power spectrum, including event-related desynchronization (ERD) and synchronization (ERS)^19^. ERD is a decrease in the neural rhythmic activity of a particular frequency when an event occurs^20^, whereas ERS is increased rhythmic activity, which represents the synchronization in the cortical response. In terms of EEG oscillation, participants with MDD show a stronger frontocentral theta synchronization after treatment with cognitive therapy^21^, individuals with depression have strong alpha activity, especially in the right hemisphere^22^, and people with a history of depression show less activated frontal delta oscillation^23^. Alpha oscillation shows significant difference between sub-threshold depression and healthy subjects during emotional facial expression^24^.

The above studies demonstrate the presence of biased cognition and differential neural activity in patients with MDD. According to Beck’s cognitive model of depression^1^, depression has an effect on schema, which leads to distorted perceptions of information about the external world. The schema of depression causes biased attention, biased processing, and biased memory, which therefore worsen the depressive situation^1^. Three competing theories—positive attenuation, negative potentiation, and emotion context insensitivity—are used to explain the distorted perception^25^. Sub-threshold depression individuals exhibited difficulties of negative emotion inhibition^26^. Individuals with depression often exhibit poor inhibition of negative stimuli and struggle to disengage from negative and neutral stimuli, demonstrating a bias in attention^27^.When exposed to happy, neutral, and sad stimuli, depressive individuals showed a lower reaction to affective stimulation compared with healthy individuals^28^. However, it remains unclear how different depressive states in healthy individuals affect neural activities in emotional information processing. Therefore, in the present study, we used negative, neutral, and positive images to investigate the different influences of various emotional stimuli. We thus investigated the different responses of healthy individuals to visual stimuli and, based on previous theories concerning the continuum between sD states and MDD, we hypothesized that this continuity might be present in the neural activities of healthy individuals.

In this study, we investigated whether the attentional bias could be detected by reaction time during the perception of image stimuli and whether there were significant differences in neural activities among healthy individuals with different depressive states during this procedure. We used the Beck Depression Inventory-Second Edition (BDI-II) to evaluate participants’ depressive states and the International Affective Picture System (IAPS) as the external visual stimulus. We grouped the IAPS images into negative, neutral, and positive categories and randomly presented one image per trial, with participants required to provide an evaluation score as soon as possible during the presentation. The image reaction time and EEG activity were recorded during the experiment. We investigated whether participants with different depressive states have attentional systems with various degrees of impairment and whether participants with higher depressive states have slower reaction times, smaller P300 amplitudes, stronger alpha oscillations, and weaker delta and theta oscillations compared with participants with lower depressive states.

## Results

### Behavioral results

**Figure 1:**
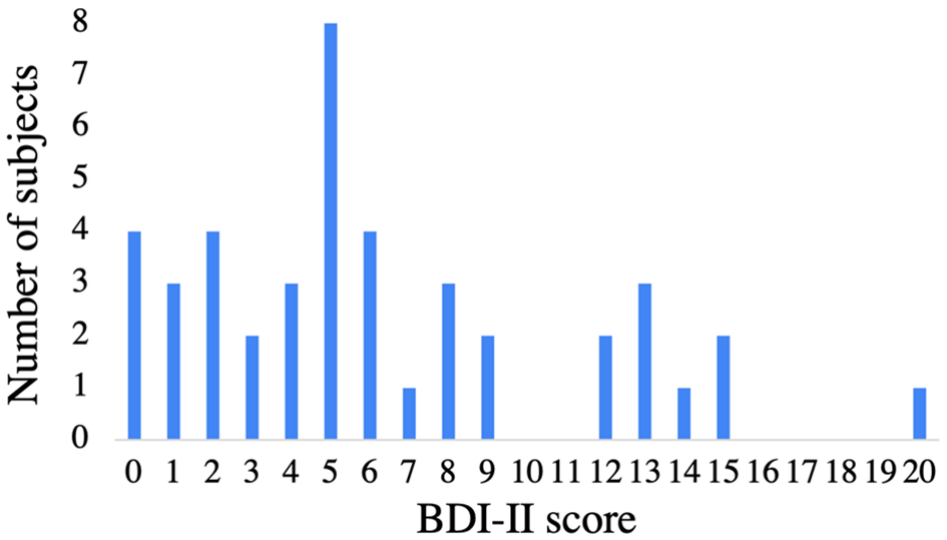
Number of subjects by BDI-II score.

The present study enrolled 43 participants, and the distribution of their depressive states is shown in Fig. 1. The participants’ depressive states were evaluated with the BDI-II. Although the maximum score for the BDI-II was 63, the maximum score for this experiment was 20 because the participants were healthy. Based on previous research, a score of 9 is commonly utilized to delineate the threshold for none to minimal depression, whereas the specific cutoffs of BDI-II 9 and 21 are applied in examining the continuity of depression symptoms, with scores exceeding 21 aligning closely with the clinical cluster^29^. The mean average BDI-II score for individuals identified as devoid of depressive tendencies at 4, thereby current research set the low depressive state as a score less than 4 (from 0 to 3)^30^. Therefore, we classified participants with scores between 0 and 3 into the low depressive state group (n = 13), those with scores between 4 and 9 into the middle depressive state group (n = 21), and those with scores between 10 and 20 into the high depressive state group (n = 9).

**Figure 2:**
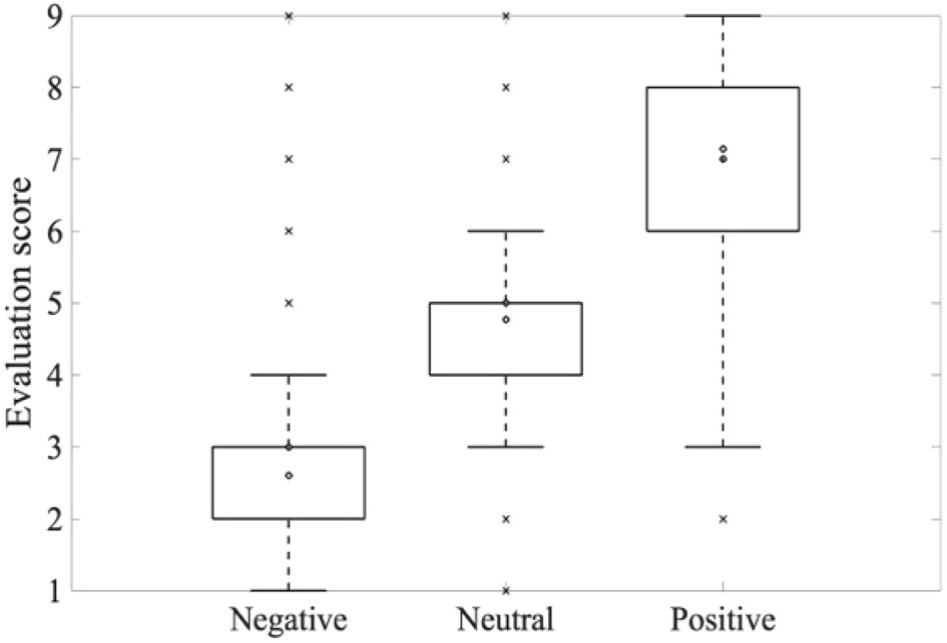
Evaluation score by image valence.

In each experimental trial, participants were asked to evaluate the pleasant and unpleasant impressions obtained from the images by pressing the numeric keys on the keyboard; the images were rated on a scale of 1 to 9, with 1 being pleasant and 9 being unpleasant. The distribution of the evaluation scores of the images by the participants for each type of image (negative, neutral, and positive) is shown in Fig. 2. The negative images were distributed in a low score range (minimum, 1 point; maximum, 4 points), indicating unpleasantness, while the positive images were distributed in a high score range (minimum, 3 points; maximum, 9 points), indicating pleasantness. In addition, the neutral images had more evaluations with a median score of 5 points. Thus, it can be concluded that specific emotions were correctly elicited when the IAPS images were presented as visual stimuli. Therefore, the three types of images in the current experiment showed the validity of specific evoked emotions.

**Figure 3:**
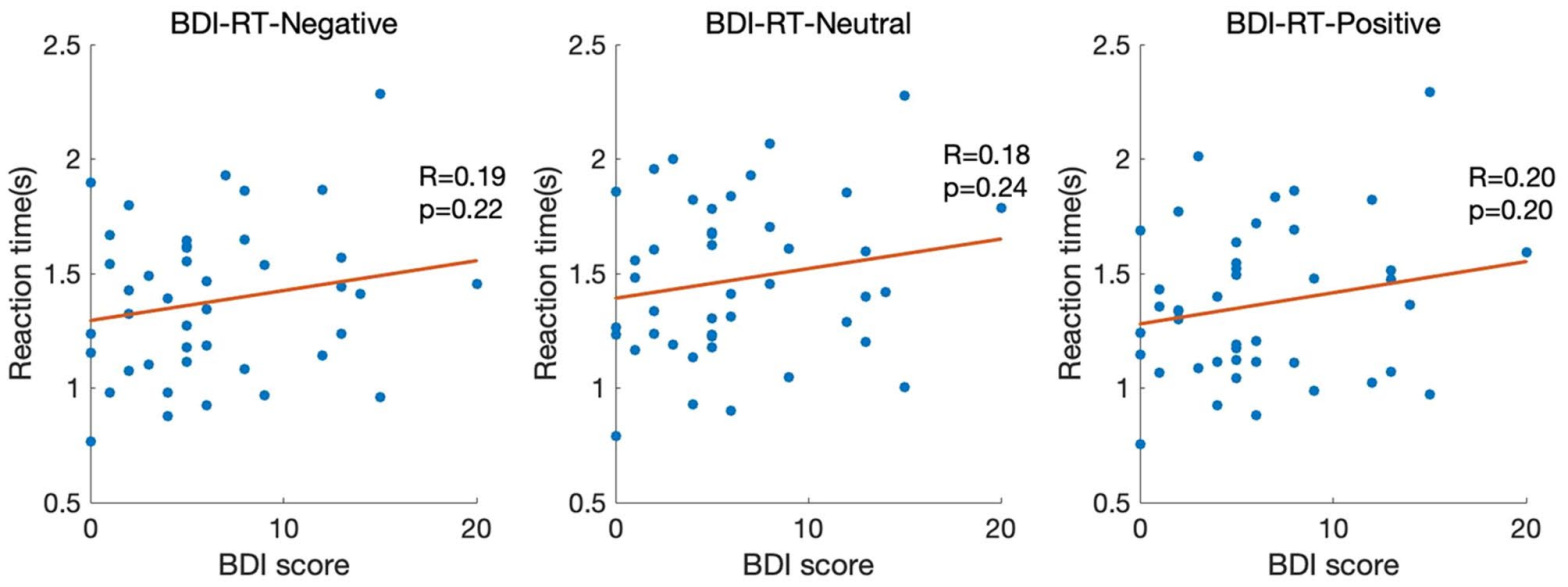
Relationship between the BDI score and reaction time. The x-axis shows the BDI score, with a higher score associated with a higher depressive state. The y-axis shows the reaction time, which was defined as the time from the appearance of the IAPS image until the subject pressed the Shift key. Each dot represents the average reaction time of one participant.

Furthermore, to determine the influence of different depressive states on reaction time, the correlation between the reaction time for each BDI-II score, rather than the three depressive state groups (high, middle, and low), is shown in Fig. 3. For all valence types of images, there were no correlations between the reaction time and BDI-II score (negative images, r = 0.191 [*p* = 0.220]; neutral images, r = 0.182 [*p* = 0.244]; positive images, r = 0.198 [*p* = 0.203]).The averaged reaction time of three groups are shown in Table 1. Statistical analysis revealed no significant influence of the groupings on reaction times (F = 0.44, *p* = 0.65).

**Table 1:** The averaged reaction time (RT) for three groups.

RT (s)	Low	Middle	High
Negative	1.34 ± 0.33	1.35 ± 0.31	1.49 ± 0.40
Neutral	1.44 ± 0.35	1.47 ± 0.34	1.54 ± 0.39
Positive	1.35 ± 0.33	1.34 ± 0.30	1.46 ± 0.42

## EEG results

- Event-related potentials

**Figure 4:**
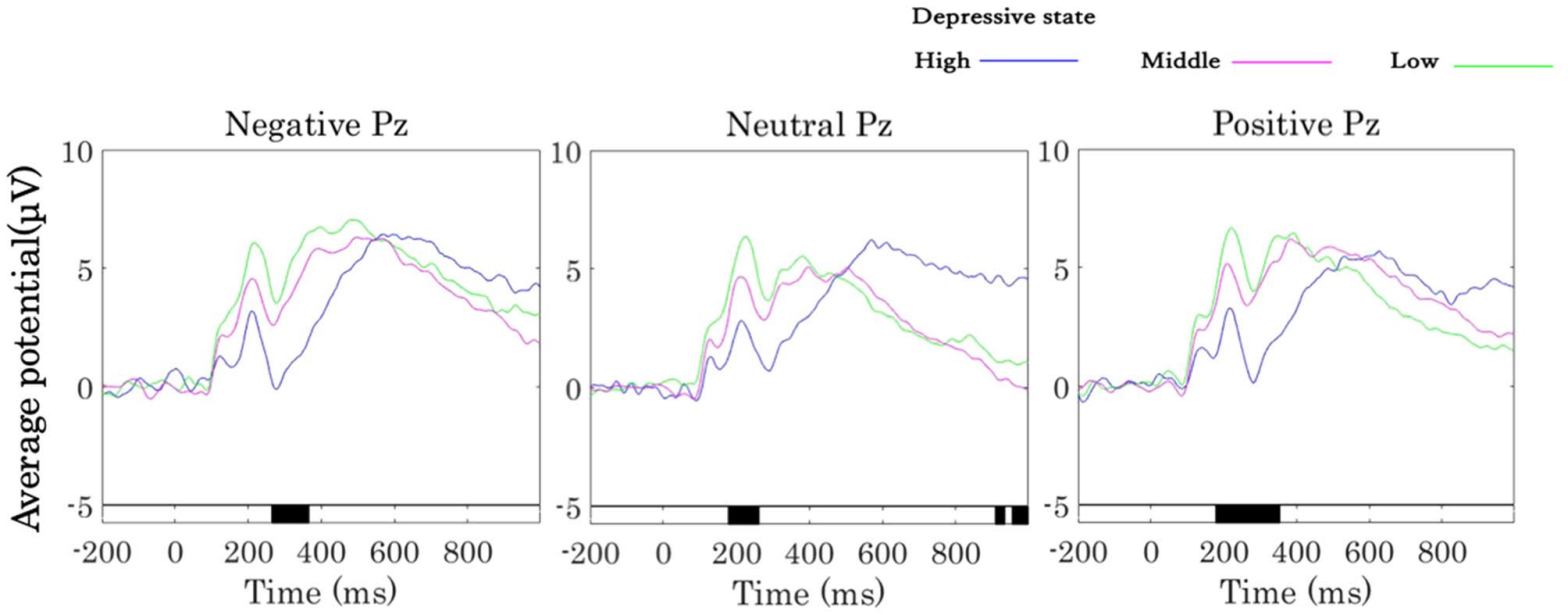
Grand averages of the event-related potential (ERP) for the three groups of IAPS images presented at Pz. On the left is the ERP figure of negative images, in the middle is the ERP figure of neutral images, and on the right is the ERP figure of positive images. The blue line represents EEG data from the high depressive state group, the pink line represents the middle depressive state group, and the green line represents the low depressive state group. The black line below the ERP figure represents the time window in which the ANOVA showed a significant difference in EEG potential according to the various depressive states.

To understand whether depressive states affect ERP amplitude during visual stimulus processing, ERP analysis was applied. ERP data were obtained for each of the three types of images (negative, neutral, and positive) and for the high, middle, and low depressive state groups. EEG signals were averaged from − 200 ms before the stimulus onset to 1000 ms after the stimulus onset. P100, which appears approximately 100 ms after visual stimulation, is considered to represent general visual information processing and is an EEG response that occurs mainly in the occipital region. The ERP results obtained at the Pz electrode, where the response is particularly evident, are shown in Fig. 4. The ERP results for high, middle, and low depressive states are remarkable at P100, P200 and P300, followed by the late positive potential (LPP), with an increase up to peak and a subsequent return to a stable state. The latencies of P100 and P200 were similar and without significant differences. For the LPP, the waveform peaked at around 500 ms in the middle and low depressive state groups, whereas the high depressive state group showed a slower response with a peak at around 600 ms. The LPPs of the middle and low depressive state groups showed larger responses to the negative and positive images but a relatively small response to the neutral images.

Analysis of variance (ANOVA) was conducted for each image type to examine whether the ERPs were affected by different depressive states (Fig. 4). The black bar attached to the time axis indicates the time range at which significant differences (FDR-p < 0.05, one-way ANOVA with repeated measures) were found. Thus, significant differences in ERPs due to depression are shown at around 250 ms to 380 ms for negative images, at around 180 ms to 300 ms for neutral images, and at around 160 ms to 380 ms for positive images.

**Figure 5:**
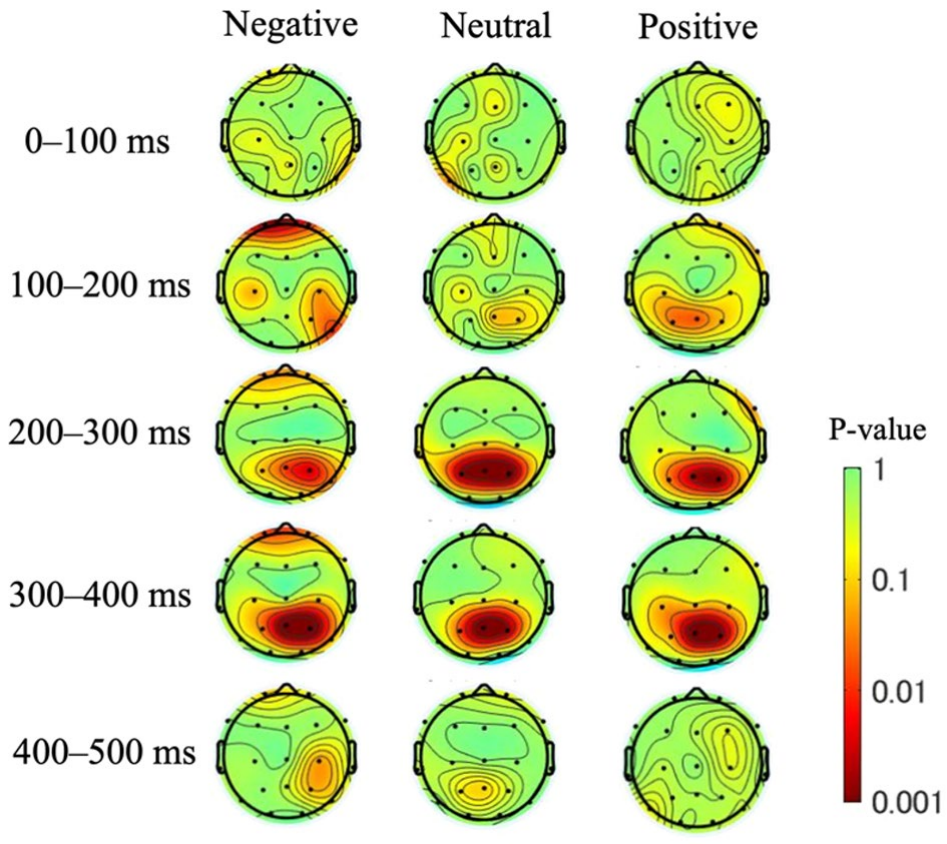
Topographic mapping of statistical differences in the ERP amplitude among the three different depressive states every 100 ms from 0 to 500 ms. The red color indicates a smaller p-value (FDR-p < 0.05, one-way ANOVA with repeated measures).

**Figure 6:**
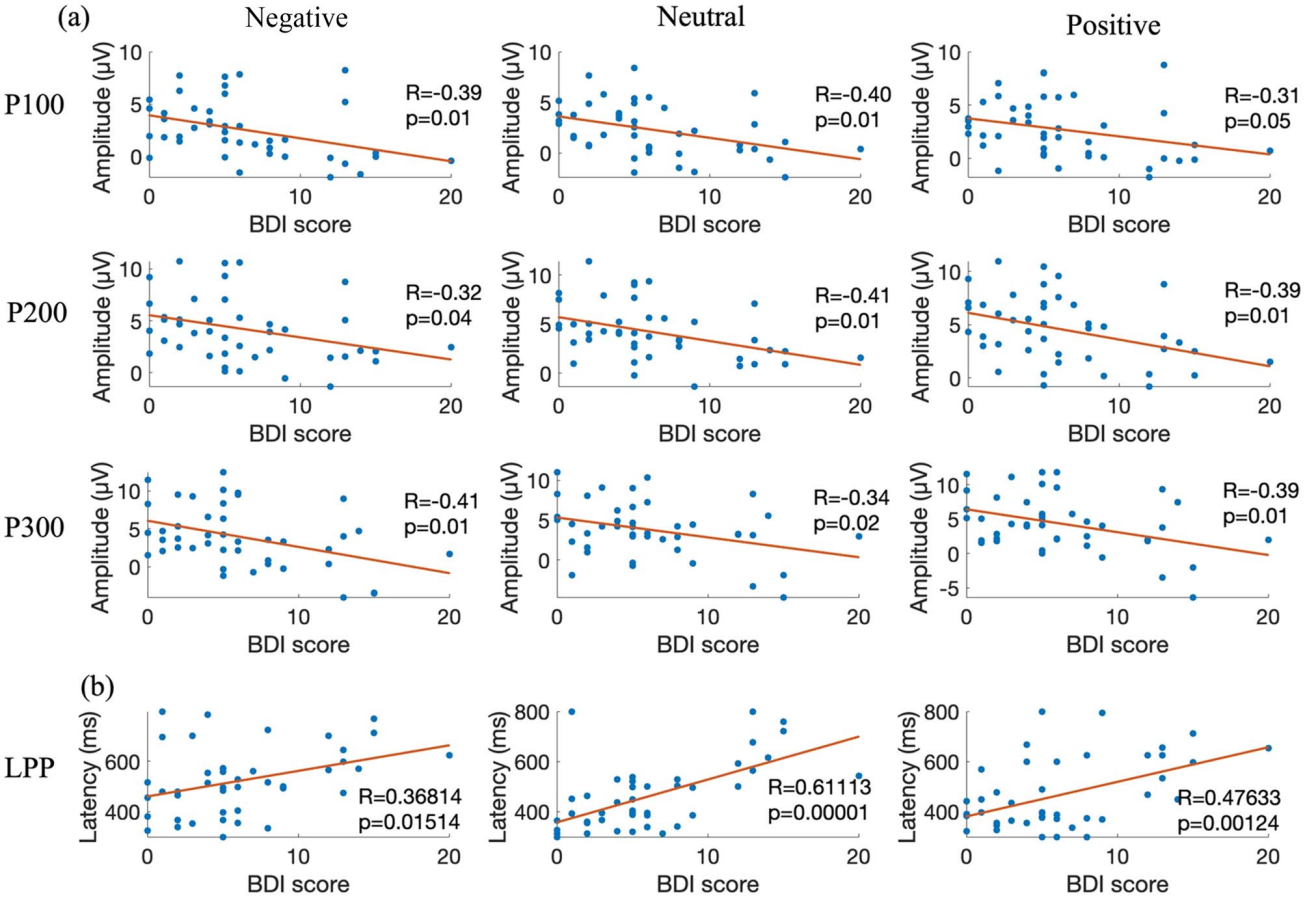
(**a**) Relationship between the BDI score and ERP amplitude. For all three groups of images (negative, neutral, and positive), the ERP amplitude were extracted and averaged and then plotted by BDI-II score. The x-axis shows the BDI score, with a higher score associated with a higher depressive state. The y-axis shows the average amplitude. The correlation of P100, P200, and P300 components are shown from up to down. (**b**) Relationship between the BDI score and ERP latency. The x-axis shows the BDI score, and the y-axis shows the peak latency.

The topographic maps of the *p*-values obtained by ANOVA (Fig. 5) were examined to determine whether the effects of different depressive states on the ERPs for each image type depended on the cerebral cortex position. Each 100-ms time interval from 0 to 500 ms after stimulus onset is shown. From 0 to 100 ms, no significant differences were observed immediately after stimulus onset. From 100 to 200 ms, significant differences were observed in the frontal region (FDR-p < 0.01, one-way ANOVA with repeated measures) in the negative image group only and in the central and parietal regions for the negative (FDR-p < 0.05, one-way ANOVA with repeated measures) and positive (FDR-p < 0.05, one-way ANOVA with repeated measures) image groups. From 200 to 400 ms after stimulus onset, in all three image groups, the p-values were small in the parietal region (FDR-p < 0.01, one-way ANOVA with repeated measures) centered on Pz and with a lateral deviation to the right hemisphere in the negative and positive image groups. In the negative image group only, there was a decrease in the degree of the significant difference in the frontal region from 100 to 400 ms. The correlation between BDI score and ERP amplitude are shown in Fig. 6a, and the correlation between BDI score and LPP latency are shown in Fig. 6b. The two-way ANOVA was applied to investigate the effect of group (low, middle and high) and stimuli (negative, neutral and positive) and their interaction effect on P100, P200 and P300. The statistical differences are shown in Table 2.

- Event-related spectrum perturbation results

**Table 2:** Statistical differences for ERP components.

ERP components	F	P
P100-group	6.36	0.002
P100-group*stimulus	0.09	0.98
P200-group	8.53	0.0003
P200-group*stimulus	0.03	0.99
P300-group	7.85	0.0006
P300-group*stimulus	0.1	0.98

**Figure 7:**
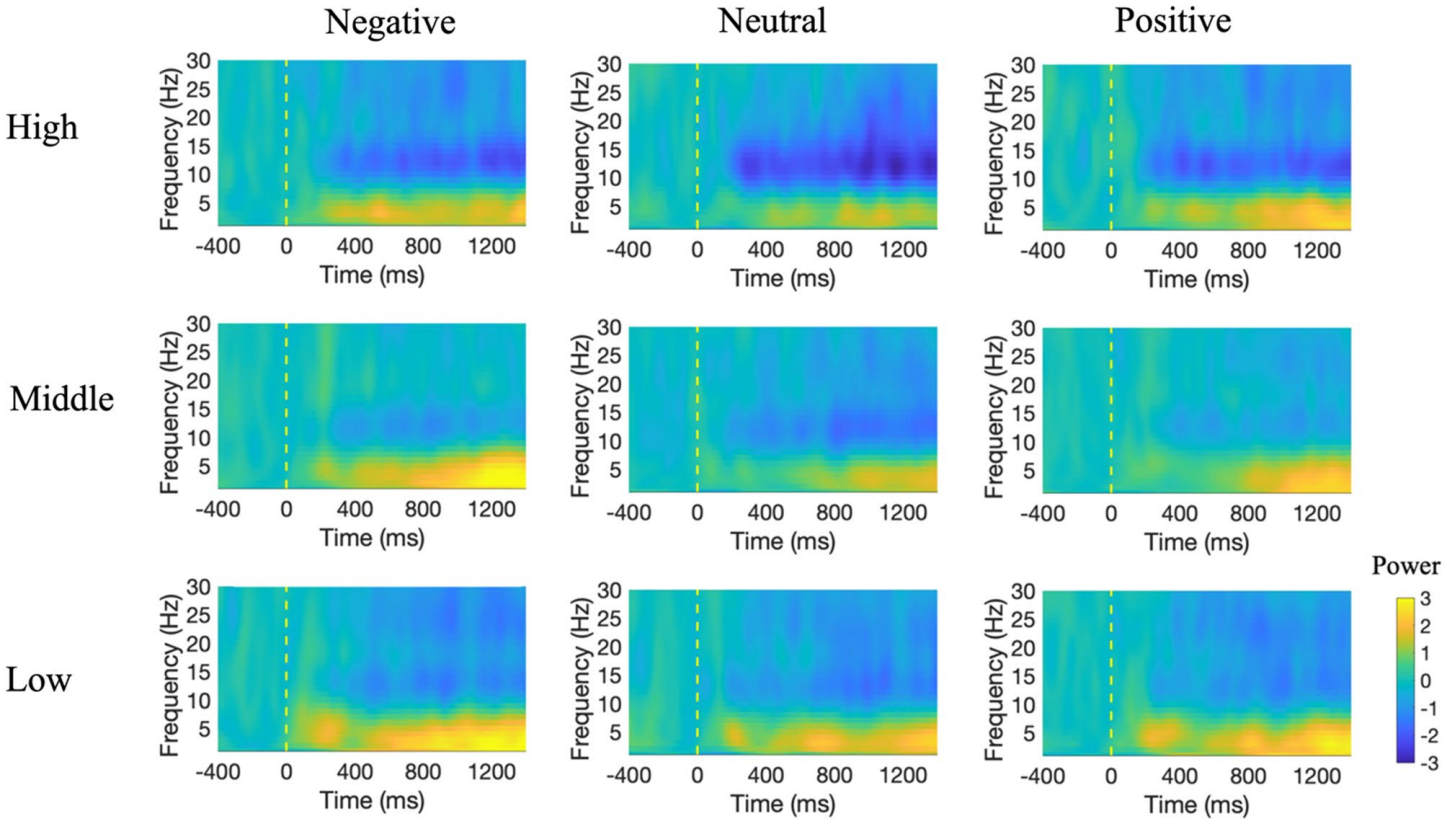
ERSP results for the three groups and three types of stimuli at Fz. The x-axis shows the time from − 400 ms to 1400 ms, where 0 ms represents the time at which the IAPS image was first displayed. The y-axis shows the frequency range from 0.5 to 30 Hz. In each figure, the yellow area is associated with synchronization and the blue area is associated with desynchronization. The three columns represent the different picture types (negative, neutral, and positive) and the three rows represent the different depressive states (high, middle, and low group).

We applied ERSP, which expresses the event-related changes in the power spectrum, to understand if depressive states affect the power changes at certain time and frequency bands during visual stimuli. As in ERP analysis, three types of images (negative, neutral, and positive) and three types of depressive state groups (high, middle, and low) were used to group and average the EEG data. The epoch length was 1800 ms (from − 400 ms before the stimulus onset to 1400 ms after it), with 400 ms before the onset used as baseline. The time–frequency result from 1 to 30 Hz obtained from Fz is shown in Fig. 7. For the ERSP result, the power increase in the delta (0–4 Hz) and theta (5–8 Hz) bands and power decrease in the alpha band (8–12 Hz) were quite pronounced from around 200 ms after stimulation onset.

**Figure 8:**
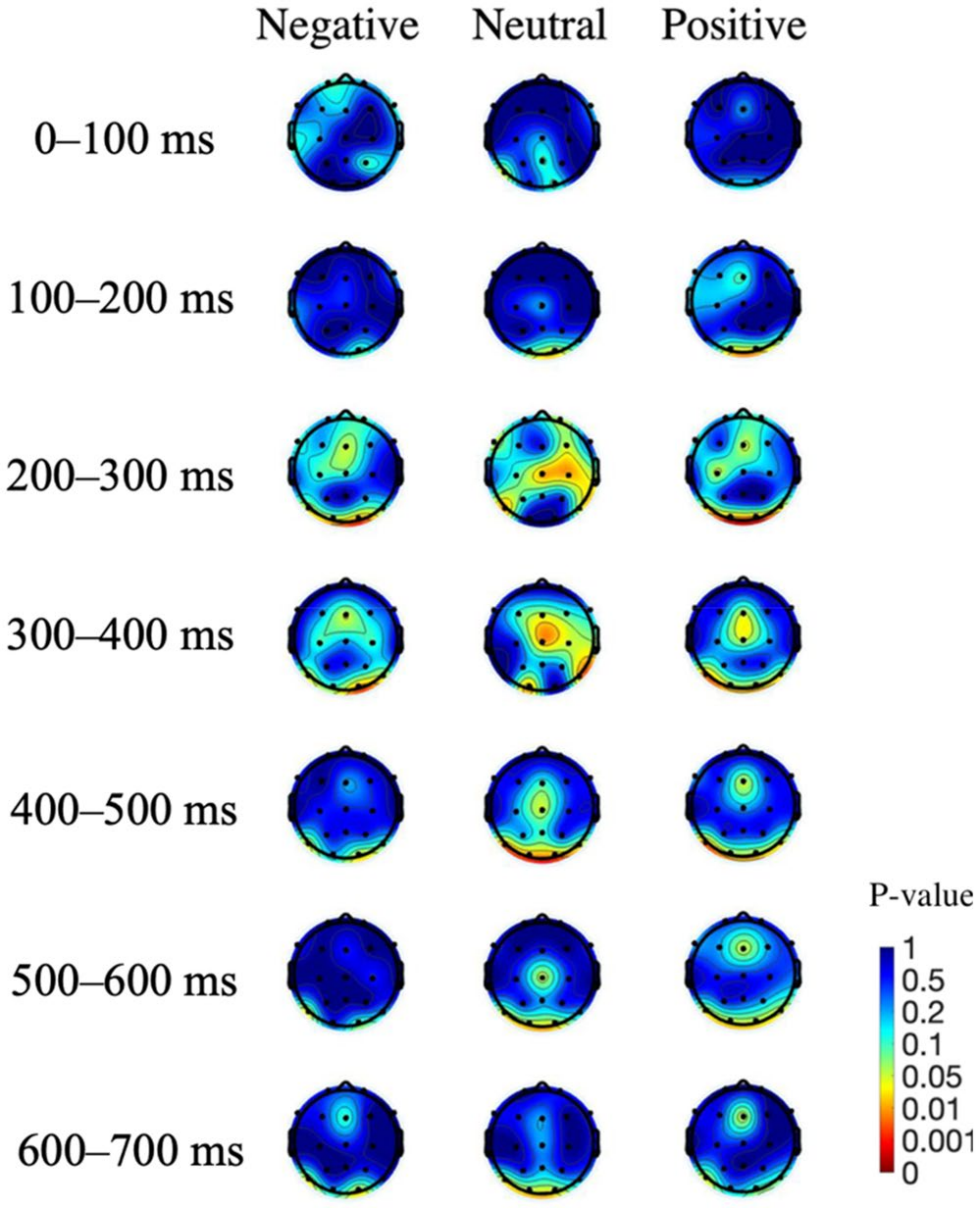
Topographic mapping of statistical differences in the ERSP amplitude of the alpha band (8–12 Hz) among the three depressive states every 100 ms from 0 to 700 ms. The red color indicates a smaller p-value (FDR-p < 0.05, one-way ANOVA with repeated measures).

As shown in Fig. 7, in terms of a decrease in amplitude, the latency of the different depressive states was similar, but activities appeared to vary in strength. The high depressive group showed a stronger decrease in the alpha band from 8 to 12 Hz, starting at around 250 ms, whereas the low and middle depressive state groups showed less of a decrease from 10 to 15 Hz. Regarding the amplitude decrease within groups for different valence pictures, the high depressive state group tended to show a greater decrease for negative and neutral images, whereas the low depressive state group showed a higher activity of the amplitude decrease for negative images. Figure 8 shows the statistical analysis result of the p-value topographic map to understand the effect of depressive states on the alpha band (8–12 Hz) ERSP. From 200 to 300 ms after stimulus onset, in all three image groups, the p-values were small in the frontal region (FDR-p < 0.01, one-way ANOVA with repeated measures) and with a lateral deviation to the left hemisphere in the frontal and temporal regions for the negative and positive image groups. From 300 to 400 ms after stimulus onset, the p-value was small in the centro-frontal region (FDR-p < 0.05 for the positive image, one-way ANOVA with repeated measures) for all three image groups. From 400 to 600 ms, there was a decrease in the degree of the significant difference in the frontal region (FDR-p < 0.05 for positive image, one-way ANOVA with repeated measures). Besides the frontal region, significant differences were also found in the occipital lobe (FDR-p < 0.01, one-way ANOVA with repeated measures) from 200 to 700 ms for the negative and positive image groups and from 300 to 700 ms for the neutral image group.

However, the result of the increase in Fig. 7 showed that the lower the amplitude increase, the more severe the depressive state. The high depressive group demonstrated a weaker amplitude increase for the delta and theta bands and shorter durations, with almost no increase produced after 600 ms, whereas individuals of low and middle depressive states showed a larger consistently sustained increase. In terms of the within-group amplitude increase for different valence pictures, the high depressive group showed a more pronounced increase for negative and neutral images, the middle depressive group showed a stronger increase for negative images, and the low depressive group showed a stronger increase for positive images.

## Discussion

This study examined neural activity differences among healthy individuals with various depressive states through analysis of ERP and time–frequency amplitude changes in the delta, theta, and alpha bands during emotional visual information processing. Regarding ERPs, a tendency emerged for the P300 amplitude to decline with an increasing depressive state for all emotional stimuli. For ERSPs, the high depressive group showed a stronger decrease in the alpha amplitude, while the low depressive group showed a stronger increase in the delta and theta amplitudes. Moreover, the three depressive state groups showed significant differences in the frontal and parietal lobes at different time periods.

For behavioral results, the correlations between the reaction time and BDI score were not significant for any of the emotional stimuli, which might be because the effect of depressive state is small for healthy participants. However, the effect of depressive states impacts EEG activity with significant differences, which suggests that EEG analysis, rather than behavioral analysis, is necessary to assess depressive states in healthy participants.

The ERP results showed that ERPs from around 100 ms to 400 ms after the presentation of the visual stimulus, mainly in P300 for the negative, neutral, and positive image groups, differed among the high, middle, and low depressive states. The ERP amplitude, which is related to attention level, tended to decrease as the depressive state increased, a finding that is consistent with our hypothesis that healthy people with a higher depressive state are impaired in their attention. Studies have shown that many ERP components are related to attention and emotional processing, such as N1, P1, P3, and LPP, and are related mainly to ERP amplitude and have little of a relationship with latency^29^. For example, the early component is associated with physical feature extraction of emotional stimuli and is found mainly in the occipital lobe, the middle latency component is related to selective processing and stimulation discrimination, and the LPP is related to memory evocation, emotional processing, and top-down processing^31^. Regarding the early ERP component, previous research has found that highly demanding visual processing tasks can be completed in less than 150 ms^32^. The positive peak P300 that appears afterward is considered to be involved in comparison, evaluation, judgment, selective attention, and updating of the cognitive context in response to stimuli^33^. The amplitude of P300 is negatively related to the degree of depression and a delay in the latency is positively related to it^34^. It has also been found that patients with MDD have a bias toward negative stimuli in both early and late ERP components, indicating the bias ranges for emotion recognition and evaluation^35^. Therefore, it is considered that the ERP responses to depression can be measured, and the ERPs elicited by external stimuli are lower in people with a high depressive state compared with the low group. This is equivalent to one of the hypotheses that the depressive state impairs the attentional system and that greater depressive state people need more time to respond to external stimuli. In addition, because the depressive state in healthy people is not qualitatively different from that of patients with MDD, but occurs on a continuum in terms of severity, this slowing of the ERP latency is also considered to occur on a continuum from people with a low depressive state to those with a high depressive state for healthy people not diagnosed with MDD.

A previous study showed that the role of P300 in regulating different emotions is more pronounced in healthy people and that the regulatory effect of LPP is impaired in individuals with high depression^36^. The P300 reflects the allocation of attention to significantly emotionally disposed stimuli, whereas the LPP is more connected with the cognitive process of distinction among emotions^14^. Compared with neutral pictures, pictures with emotional tendencies can elicit a greater LPP, which appears at around 400 ms, showing the selective processing of emotional stimuli in the brain^37^. The LPP also reflects a biased processing of self-referential stimuli^9^. Patients with generalized anxiety disorder show less attentional adjustment in response to negative stimuli, as found through the LPP^38^. In this study, the P300 amplitude decreased in the high depressive state group in response to emotional stimuli, and the LPP latency increased significantly with the increasing level of the depressive state, which might reflect the biased processing and impaired processing procedure. In addition, the topographic images showed a significant difference among depressive state groups in the frontal potentials only in the negative image group, which might suggest that the information processing is particularly affected by depressive state in the frontal lobe. ERP responses originating from external stimuli are believed to occur from about 30 ms after the presentation of visual stimuli, and comprehensive rough cognition appears to take place in the frontal cortex^39^. Regarding the early ERP component, previous research has found that highly demanding visual processing tasks can be completed in less than 150 ms^31^. This might suggest that only the negative image group with negative impressions consistent with the schema, influenced by different schema depending on the degree of the depression, will show a significant difference by depressive state.

We also explored the EEG oscillation based on high, middle, and low depressive states during emotional stimulation by time–frequency features. Previous research found that participants with high emotional intelligence exhibited less upper alpha desynchronization in emotion recognition testing^40^. People with low emotional granularity^41^, who are unable to express their emotions with discrete words, show greater alpha ERD, which is thought to be associated with greater effort to recognize affective stimuli^20^. Later-stage alpha oscillation is associated with higher cognitive functions, such as the discrimination of emotional stimuli^42^. Alpha desynchronization is associated with special tasks and increased information transmission^43^, whereas frontal alpha synchronization is related to top-down processing and creative tasks and theta synchronization is related to the evaluation of emotional stimuli^44^. In this study, we found that the higher depressive group showed a higher alpha ERD, suggesting that depressive states may be related to the degree of awareness of emotions, with high depressive state people having a more stunted perception and recognition of emotions.

Furthermore, topographic analysis revealed significant differences from 100 to 300 ms in the posterior region and left prefrontal region among the depressive state groups, with the difference found in turn at the frontal area and central area. Previous research identified increased alpha power in the frontal-parietal lobe in patients with MDD^45^. A neural mechanism for top-down visual stimulus recognition proposes that visual stimuli are first projected to the prefrontal cortex for initial recognition and then to the temporal cortex in combination with bottom-up processes, allowing for more rapid recognition^46^. The dorsolateral prefrontal cortex in the left hemisphere has also been associated with attention regulation^47^. Therefore, the results might indicate that the neural mechanisms of top-down emotion regulation in response to emotional stimuli differ among depressive state groups, and there may be a significant relationship between how they respond to negative stimuli and their cognitive schema.

In terms of delta and theta bands, we found the opposite tendency, that is, the higher the depressive state, the lower the ERS. Delta synchronization was determined to occur at an earlier time and to be involved in detecting emotional significance. Previous research found that the enhancement of theta synchronization in posterior and anterior regions is related to the degree of concentration and to the mechanisms for emotion processing^41^. In terms of emotional tendency, high emotional salience stimuli induce larger delta and theta band synchronizatio^37^. Theta synchronization in the posterior region has been correlated with evaluations of emotional salience, with scary stimuli eliciting more intense synchronization^43^. Thus, the lower delta and theta ERS may reflect a lack of attentional resources and a deficit in the detection of emotional salience in individuals with a higher depressive state compared with those with a lower depressive state.

The current experiment has some limitations. First, depression is more prevalent in the female population^48^, but about two-thirds of the participants in this experiment were men. This gender imbalance may not allow for replication across the female population, and future research will have to improve the generalizability. Furthermore, although the previous study validated the effectiveness of using the BDI-II score to group participants^2^, it was difficult to conclude that there was a sufficient sample for each group, and future studies will need to increase the sample to identify significant relationships in the behavioral data.

In this study, we found that the amplitude and latency of ERP components and the strength of alpha desynchronization and theta synchronization in response to emotional visual stimuli were modulated by depressive state in healthy participants. Thus, we suggest that depressive states in healthy individuals may also modulate early cognitive and later attentional control, as well as emotion discrimination processes. This neural circuit based on the response between frontal and parietal regions may contribute to the understanding of emotion-cognition regulation in healthy individuals. This circuit contains both top-down and bottom-up bidirectional neural circuits, reflecting the formation of depressive states accompanied by schema reinforcement and diminished attentional control. The finding of these neural responses associated with depressive states is informative and can be used as one of the parameters to assess the evaluation of depressive states in healthy individuals and might serve as an early indicator of the possibility of MDD.

## Methods

### Participants

The experiment recruited 46 participants (16 women and 30 men; mean age, 22 years) who had not received a depression diagnosis and were not receiving medication. This study was approved by the Ethical Review of Human Subject Research of the Tokyo Institute of Technology (Approval Number: A20202) and Ethical Review of Human Research of Tokai University (Approval Number: 21134). All methods were performed in accordance with relevant guidelines and regulations, and informed consent was obtained from all subjects. Data from three of the participants were removed due to incomplete data or because they had significantly different responses from other participants in the experiment.

The BDI-II was used to evaluate the participants’ depressive states. The BDI-II contains 21 questions related to the respondents’ life and psychological conditions in the last 2 weeks. Each question has four options, with points scored from 0 to 3 and a higher score corresponding to a stronger state of depression. Thus, the depression is rated from 0 to 63 (the lower the overall score, the lower the depressive state). Participants answered all of the questions on the Google Form before the EEG recording experiment, and their depressive state was scored and evaluated. Because the participants in this study were healthy individuals who had not been diagnosed with a psychiatric disorder, including depression, there were no participants with significantly high BDI-II scores and the score variation was considered to be small (mean, 6.413; standard deviation, 4.773).

### Visual stimuli

In this experiment, the IAPS was used as the external stimulus presented to the participants during the experiment^49^. The IAPS is a set of affective images comprising 956 different images of, for example, objects, scenes, plants and animals, and human faces. It is widely used in affective and cognitive experiments worldwide, mainly to induce emotions. The IAPS is accompanied by three kinds of emotional values that have been evaluated by the makers of IAPS in their emotional evaluation experiments for each image, and users can select the most suitable image for their experiments using these values as a reference^50^. The three emotional parameters are valence, arousal, and dominance. In this experiment, the valence parameter, which represents pleasantness-unpleasantness, was used to select the image stimuli. The selected images were classified into three groups according to valence: negative (unpleasant images), neutral (image without strong emotional induction), and positive (pleasant images)^51^. Images with a low valence value (1.66–2.93) were considered negative images with unpleasant impressions, images with a medium valence value (4.50–5.01) were considered neutral images without particularly strong emotional induction, and images with a high valence value (7.09–8.34) were considered positive images with pleasant impressions. In total, 60 images of each type were selected and used in the experiment.

### Experimental design

**Figure 9:**
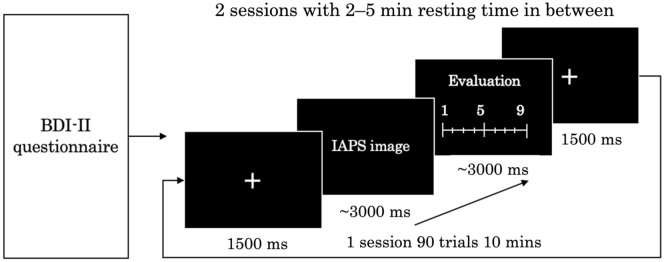
The protocol of the experiment.

The experiment was performed as shown in Fig. 9. First, depression was assessed using the BDI-II, which was followed by placement of the EEG electrodes, explanation of the experimental procedure, and a practice session. Then, the experiment involving the presentation of IAPS images was performed in two sessions with a break in between. In each session, 90 IAPS images were presented randomly, and the total time of the two sessions including breaks was about 30 min. As shown in Fig. 9, the experimental process in each trial was divided into three stages. (1) First, a “ + ” mark was displayed in the center of the screen monitor for 1.5 s to guide the participants’ gaze, during which time they waited without moving their bodies. (2) After that, an IAPS image was randomly displayed, and the participants recognized what the image was and switched to the next evaluation screen by pressing the Shift key on the keyboard if they could rate their impressions as pleasant or unpleasant. The Shift key was to be pressed as soon as possible within 3 s after the image was displayed and, after 3 s, the evaluation screen was automatically switched to the next stage. (3) In the evaluation stage, the participants rated the pleasant and unpleasant impressions obtained from the images on a scale of 1 to 9 by pressing the numeric keys from 1 to 9 on the keyboard, with 1 being pleasant and 9 being unpleasant. The number keys were to be pressed as soon as possible within 3 s after the evaluation screen was displayed and, after 3 s, the screen was automatically switched to the next trial. The experiments were conducted in a bright soundproof room, with the participants sitting comfortably in a chair and keeping their heads on a chin rest to prevent head movement.

### Data measurement and analysis

We recorded EEG data with an EEG amplifier (Polymate Pro MP6100, Miyuki Giken Co., Tokyo, Japan) to acquire signals based on the International 10–20 system from 21 electrodes, including two electrodes measuring the vertical electrooculogram (VEOG) and horizontal electrooculogram (HEOG), with a sampling rate of 500 Hz. The impedance of the electrodes was below 50 kΩ, as recommended.

- Behavioral data

We used Python scripts to write the whole experiment, using an Arduino to connect the EEG amplifier (Polymate Pro MP6100) to the PC displaying the experimental procedure so that we could record the exact time at which each experimental stage started. The reaction time, which was defined as the time from the appearance of the IAPS image until the individual pressed the Shift key, and the evaluation score for the image were recorded in a text file. Trials with a reaction time equaling 3000 ms were eliminated, and the remaining data were used for behavioral result analysis. Polynomial curve fitting^52^ and polynomial evaluation^53^ were applied to fit a simple linear regression model to the sets of BDI-RT data and BDI evaluation data.

- EEG data

The raw data were referenced online to AFz and re-referenced to the average of all electrodes in pre-processing. Eye movement components that were similar to two EOG recordings were removed by the Infomax ICA algorithm. The EEG analysis was based on a MATLAB script and EEGLAB toolbox, as well as its plugin ERPLAB. Regarding the pre-processing of EEG data, a bandpass filter was applied from 0.5 to 30 Hz.

### Analysis method for ERP

The ERP analysis was applied from − 200 to 1000 ms with respect to the time when the IAPS image appeared. The ERP amplitude of each epoch was extracted by image type (negative, neutral, and positive) and then divided into three depressive state groups (high, middle, and low depressive) based on BDI-II score. One-way ANOVA was applied to each 100-ms time period from 0 to 500 ms and each image type to investigate if there were significant differences among the three depressive state groups. For statistical analysis, we assessed 19 channels, applying a correction for multiple comparisons to examine the primary effect of the depressive state. Due to the significant differences and a representative pattern in the waveform, our focus was narrowed to the Pz channel. The calculation of mean amplitudes was conducted within time windows centered at the peak latencies of ERP components in the grand-mean waveforms, utilizing shorter window lengths for early components and longer lengths for late components. Our analysis encompassed the average amplitudes of the parietal P100, P200, and P300. Specifically, the P100 amplitude was calculated as the average amplitude at Pz between 150 to 180 ms following the onset of words. The P200 amplitude was determined in the timeframe of 180 to 220 ms post-stimulus at Pz, and the P300 amplitude was ascertained as the average amplitude at Pz between 250 to 400 ms post-stimulus. The LPP latency was calculated as the time at which the relative-to-baseline peak value at Pz between 300 to 800 ms post stimulus.

### Analysis method for ERSP

For the time–frequency domain feature, we applied ERSP analysis. To compute it, the signal of each trial was submitted to a Morlet wavelet transform, and the baseline power from − 200 ms to 0 ms was subtracted from the spectral power after stimulus onset. Then, the results were averaged based on the three types of images of different valences (negative, neutral, and positive) and different depressive states. One-way ANOVA was conducted for each electrode and every 100 ms. The time interval was set to 100 ms from 0 to 700 ms after stimulus onset, and the average ERSP strength of the alpha band (8–12 Hz) was extracted to investigate if there were significant differences among the three depressive state groups. The statistical analysis was against 19 channels with correction of multiple comparisons to test the effect of depressive state. Because of the significant difference could be mainly found in frontal area, we focused on Fz channel for ERSP analysis. The selection of electrodes was a post-hoc decision based on our data.

## Data Availability

The datasets analyzed during the current study are not publicly available due to ethical concerns but are available from the corresponding author on reasonable request.
